# Supplementary material for: HIVprotI: an integrated web based platform for prediction and design of HIV proteins inhibitors
Source: J Cheminform. 2018 Mar 9;10:12. doi: 10.1186/s13321-018-0266-y (PMC5845081; doi:10.1186/s13321-018-0266-y)
Supplement: Supplementary file 2 — Additional file 2. Source code of HIVProtI web server. [file 13321_2018_266_MOESM2_ESM.zip › HIVprotI_Source-code/hivprotI/weka-3-6-6/doc/weka/gui/ResultHistoryPanel.html]

ResultHistoryPanel


---


|  |  |  |
| --- | --- | --- |
| **Overview** \| **Package** \| **Class** \| **Tree** \| **Deprecated** \| **Index** \| **Help** \| **Weka's home** | |  |
| **PREV CLASS**   **NEXT CLASS** | **FRAMES**    **NO FRAMES**     **All Classes** |  |
| SUMMARY: NESTED \| FIELD \| CONSTR \| METHOD | DETAIL: FIELD \| CONSTR \| METHOD |  |


---


## weka.gui Class ResultHistoryPanel

```
java.lang.Object
  java.awt.Component
      java.awt.Container
          javax.swing.JComponent
              javax.swing.JPanel
                  weka.gui.ResultHistoryPanel
```

**All Implemented Interfaces:**: java.awt.image.ImageObserver, java.awt.MenuContainer, java.io.Serializable, javax.accessibility.Accessible

---

``` public class ResultHistoryPanel extends javax.swing.JPanel ```

A component that accepts named stringbuffers and displays the name in a list
box. When a name is right-clicked, a frame is popped up that contains
the string held by the stringbuffer. Optionally a text component may be
provided that will have it's text set to the named result text on a
left-click.

**Version:**
:   $Revision: 7059 $

**Author:**
:   Len Trigg (trigg@cs.waikato.ac.nz)

**See Also:**: Serialized Form

---

| **Nested Class Summary** | |
| --- | --- |
| `static class` | `ResultHistoryPanel.RKeyAdapter`             Extension of KeyAdapter that implements Serializable. |
| `static class` | `ResultHistoryPanel.RMouseAdapter`             Extension of MouseAdapter that implements Serializable. |

**Nested classes/interfaces inherited from class javax.swing.JComponent**

`javax.swing.JComponent.AccessibleJComponent`

**Nested classes/interfaces inherited from class java.awt.Component**

`java.awt.Component.BaselineResizeBehavior`


**Field Summary**

**Fields inherited from class javax.swing.JComponent**

`TOOL_TIP_TEXT_KEY, UNDEFINED_CONDITION, WHEN_ANCESTOR_OF_FOCUSED_COMPONENT, WHEN_FOCUSED, WHEN_IN_FOCUSED_WINDOW`

**Fields inherited from class java.awt.Component**

`BOTTOM_ALIGNMENT, CENTER_ALIGNMENT, LEFT_ALIGNMENT, RIGHT_ALIGNMENT, TOP_ALIGNMENT`

**Fields inherited from interface java.awt.image.ImageObserver**

`ABORT, ALLBITS, ERROR, FRAMEBITS, HEIGHT, PROPERTIES, SOMEBITS, WIDTH`


**Constructor Summary**

`ResultHistoryPanel(javax.swing.text.JTextComponent text)`
  
          Create the result history object


| **Method Summary** | |
| --- | --- |
| `void` | `addObject(java.lang.String name, java.lang.Object o)`             Adds an object to the results list |
| `void` | `addResult(java.lang.String name, java.lang.StringBuffer result)`             Adds a new result to the result list. |
| `void` | `clearResults()`             Removes all of the result buffers from the history. |
| `javax.swing.JList` | `getList()`             Gets the JList used by the results list |
| `java.lang.String` | `getNameAtIndex(int index)`             Gets the name of theitem in the list at the specified index |
| `java.lang.StringBuffer` | `getNamedBuffer(java.lang.String name)`             Gets the named buffer |
| `java.lang.Object` | `getNamedObject(java.lang.String name)`             Get the named object from the list |
| `java.lang.StringBuffer` | `getSelectedBuffer()`             Gets the buffer associated with the currently selected item in the list. |
| `java.lang.String` | `getSelectedName()`             Get the name of the currently selected item in the list |
| `java.lang.Object` | `getSelectedObject()`             Gets the object associated with the currently selected item in the list. |
| `javax.swing.ListSelectionModel` | `getSelectionModel()`             Gets the selection model used by the results list. |
| `static void` | `main(java.lang.String[] args)`             Tests out the result history from the command line. |
| `void` | `openFrame(java.lang.String name)`             Opens the named result in a separate frame. |
| `void` | `removeResult(java.lang.String name)`             Removes one of the result buffers from the history. |
| `void` | `setHandleRightClicks(boolean tf)`             Set whether the result history list should handle right clicks or whether the parent object will handle them. |
| `void` | `setSingle(java.lang.String name)`             Sets the single-click display to view the named result. |
| `void` | `updateResult(java.lang.String name)`             Tells any component currently displaying the named result that the contents of the result text in the StringBuffer have been updated. |

**Methods inherited from class javax.swing.JPanel**

`getAccessibleContext, getUI, getUIClassID, setUI, updateUI`

**Methods inherited from class javax.swing.JComponent**

`addAncestorListener, addNotify, addVetoableChangeListener, computeVisibleRect, contains, createToolTip, disable, enable, firePropertyChange, firePropertyChange, firePropertyChange, getActionForKeyStroke, getActionMap, getAlignmentX, getAlignmentY, getAncestorListeners, getAutoscrolls, getBaseline, getBaselineResizeBehavior, getBorder, getBounds, getClientProperty, getComponentPopupMenu, getConditionForKeyStroke, getDebugGraphicsOptions, getDefaultLocale, getFontMetrics, getGraphics, getHeight, getInheritsPopupMenu, getInputMap, getInputMap, getInputVerifier, getInsets, getInsets, getListeners, getLocation, getMaximumSize, getMinimumSize, getNextFocusableComponent, getPopupLocation, getPreferredSize, getRegisteredKeyStrokes, getRootPane, getSize, getToolTipLocation, getToolTipText, getToolTipText, getTopLevelAncestor, getTransferHandler, getVerifyInputWhenFocusTarget, getVetoableChangeListeners, getVisibleRect, getWidth, getX, getY, grabFocus, isDoubleBuffered, isLightweightComponent, isManagingFocus, isOpaque, isOptimizedDrawingEnabled, isPaintingForPrint, isPaintingTile, isRequestFocusEnabled, isValidateRoot, paint, paintImmediately, paintImmediately, print, printAll, putClientProperty, registerKeyboardAction, registerKeyboardAction, removeAncestorListener, removeNotify, removeVetoableChangeListener, repaint, repaint, requestDefaultFocus, requestFocus, requestFocus, requestFocusInWindow, resetKeyboardActions, reshape, revalidate, scrollRectToVisible, setActionMap, setAlignmentX, setAlignmentY, setAutoscrolls, setBackground, setBorder, setComponentPopupMenu, setDebugGraphicsOptions, setDefaultLocale, setDoubleBuffered, setEnabled, setFocusTraversalKeys, setFont, setForeground, setInheritsPopupMenu, setInputMap, setInputVerifier, setMaximumSize, setMinimumSize, setNextFocusableComponent, setOpaque, setPreferredSize, setRequestFocusEnabled, setToolTipText, setTransferHandler, setVerifyInputWhenFocusTarget, setVisible, unregisterKeyboardAction, update`

**Methods inherited from class java.awt.Container**

`add, add, add, add, add, addContainerListener, addPropertyChangeListener, addPropertyChangeListener, applyComponentOrientation, areFocusTraversalKeysSet, countComponents, deliverEvent, doLayout, findComponentAt, findComponentAt, getComponent, getComponentAt, getComponentAt, getComponentCount, getComponents, getComponentZOrder, getContainerListeners, getFocusTraversalKeys, getFocusTraversalPolicy, getLayout, getMousePosition, insets, invalidate, isAncestorOf, isFocusCycleRoot, isFocusCycleRoot, isFocusTraversalPolicyProvider, isFocusTraversalPolicySet, layout, list, list, locate, minimumSize, paintComponents, preferredSize, printComponents, remove, remove, removeAll, removeContainerListener, setComponentZOrder, setFocusCycleRoot, setFocusTraversalPolicy, setFocusTraversalPolicyProvider, setLayout, transferFocusBackward, transferFocusDownCycle, validate`

**Methods inherited from class java.awt.Component**

`action, add, addComponentListener, addFocusListener, addHierarchyBoundsListener, addHierarchyListener, addInputMethodListener, addKeyListener, addMouseListener, addMouseMotionListener, addMouseWheelListener, bounds, checkImage, checkImage, contains, createImage, createImage, createVolatileImage, createVolatileImage, dispatchEvent, enable, enableInputMethods, firePropertyChange, firePropertyChange, firePropertyChange, firePropertyChange, firePropertyChange, getBackground, getBounds, getColorModel, getComponentListeners, getComponentOrientation, getCursor, getDropTarget, getFocusCycleRootAncestor, getFocusListeners, getFocusTraversalKeysEnabled, getFont, getForeground, getGraphicsConfiguration, getHierarchyBoundsListeners, getHierarchyListeners, getIgnoreRepaint, getInputContext, getInputMethodListeners, getInputMethodRequests, getKeyListeners, getLocale, getLocation, getLocationOnScreen, getMouseListeners, getMouseMotionListeners, getMousePosition, getMouseWheelListeners, getName, getParent, getPeer, getPropertyChangeListeners, getPropertyChangeListeners, getSize, getToolkit, getTreeLock, gotFocus, handleEvent, hasFocus, hide, imageUpdate, inside, isBackgroundSet, isCursorSet, isDisplayable, isEnabled, isFocusable, isFocusOwner, isFocusTraversable, isFontSet, isForegroundSet, isLightweight, isMaximumSizeSet, isMinimumSizeSet, isPreferredSizeSet, isShowing, isValid, isVisible, keyDown, keyUp, list, list, list, location, lostFocus, mouseDown, mouseDrag, mouseEnter, mouseExit, mouseMove, mouseUp, move, nextFocus, paintAll, postEvent, prepareImage, prepareImage, remove, removeComponentListener, removeFocusListener, removeHierarchyBoundsListener, removeHierarchyListener, removeInputMethodListener, removeKeyListener, removeMouseListener, removeMouseMotionListener, removeMouseWheelListener, removePropertyChangeListener, removePropertyChangeListener, repaint, repaint, repaint, resize, resize, setBounds, setBounds, setComponentOrientation, setCursor, setDropTarget, setFocusable, setFocusTraversalKeysEnabled, setIgnoreRepaint, setLocale, setLocation, setLocation, setName, setSize, setSize, show, show, size, toString, transferFocus, transferFocusUpCycle`

**Methods inherited from class java.lang.Object**

`equals, getClass, hashCode, notify, notifyAll, wait, wait, wait`

**Constructor Detail**

### ResultHistoryPanel

```
public ResultHistoryPanel(javax.swing.text.JTextComponent text)
```

:   Create the result history object

    **Parameters:**: `text` - the optional text component for single-click display


**Method Detail**

### addResult

```
public void addResult(java.lang.String name,
                      java.lang.StringBuffer result)
```

:   Adds a new result to the result list.

    :   **Parameters:**: `name` - the name to associate with the result: `result` - the StringBuffer that contains the result text

---


### removeResult

```
public void removeResult(java.lang.String name)
```

:   Removes one of the result buffers from the history. Any windows currently
    displaying the contents of the buffer are not affected.

    :   **Parameters:**: `name` - the name of the buffer to remove.

---


### clearResults

```
public void clearResults()
```

:   Removes all of the result buffers from the history. Any windows currently
    displaying the contents of the buffer are not affected.

---


### addObject

```
public void addObject(java.lang.String name,
                      java.lang.Object o)
```

:   Adds an object to the results list

    :   **Parameters:**: `name` - the name to associate with the object: `o` - the object

---


### getNamedObject

```
public java.lang.Object getNamedObject(java.lang.String name)
```

:   Get the named object from the list

    :   **Parameters:**: `name` - the name of the item to retrieve the stored object for **Returns:**: the object or null if there is no object at this index

---


### getSelectedObject

```
public java.lang.Object getSelectedObject()
```

:   Gets the object associated with the currently
    selected item in the list.

    :   **Returns:**: the object or null if there is no object corresponding to the current selection in the list

---


### getNamedBuffer

```
public java.lang.StringBuffer getNamedBuffer(java.lang.String name)
```

:   Gets the named buffer

    :   **Returns:**: the buffer or null if there are no items in the list

---


### getSelectedBuffer

```
public java.lang.StringBuffer getSelectedBuffer()
```

:   Gets the buffer associated with the currently
    selected item in the list.

    :   **Returns:**: the buffer or null if there are no items in the list

---


### getSelectedName

```
public java.lang.String getSelectedName()
```

:   Get the name of the currently selected item in the list

    :   **Returns:**: the name of the currently selected item or null if no item selected

---


### getNameAtIndex

```
public java.lang.String getNameAtIndex(int index)
```

:   Gets the name of theitem in the list at the specified index

    :   **Returns:**: the name of item or null if there is no item at that index

---


### setSingle

```
public void setSingle(java.lang.String name)
```

:   Sets the single-click display to view the named result.

    :   **Parameters:**: `name` - the name of the result to display.

---


### openFrame

```
public void openFrame(java.lang.String name)
```

:   Opens the named result in a separate frame.

    :   **Parameters:**: `name` - the name of the result to open.

---


### updateResult

```
public void updateResult(java.lang.String name)
```

:   Tells any component currently displaying the named result that the
    contents of the result text in the StringBuffer have been updated.

    :   **Parameters:**: `name` - the name of the result that has been updated.

---


### getSelectionModel

```
public javax.swing.ListSelectionModel getSelectionModel()
```

:   Gets the selection model used by the results list.

    :   **Returns:**: a value of type 'ListSelectionModel'

---


### getList

```
public javax.swing.JList getList()
```

:   Gets the JList used by the results list

    :   **Returns:**: the JList

---


### setHandleRightClicks

```
public void setHandleRightClicks(boolean tf)
```

:   Set whether the result history list should handle right clicks
    or whether the parent object will handle them.

    :   **Parameters:**: `tf` - false if parent object will handle right clicks

---


### main

```
public static void main(java.lang.String[] args)
```

:   Tests out the result history from the command line.

    :   **Parameters:**: `args` - ignored


---


|  |  |  |
| --- | --- | --- |
| **Overview** \| **Package** \| **Class** \| **Tree** \| **Deprecated** \| **Index** \| **Help** \| **Weka's home** | |  |
| **PREV CLASS**   **NEXT CLASS** | **FRAMES**    **NO FRAMES**     **All Classes** |  |
| SUMMARY: NESTED \| FIELD \| CONSTR \| METHOD | DETAIL: FIELD \| CONSTR \| METHOD |  |


---
